# Spontaneous Pregnancy after 4D-Hysterosalpingo-Sonography (HyCoSy) in the Same Menstrual Cycle: A Case Report and an Updating Review of the Current Literature regarding the Positive Impact of Tubal Flushing Effect on Fertility

**DOI:** 10.1155/2024/7774854

**Published:** 2024-06-11

**Authors:** Francesco Bisogni, Francesco Galanti, Serena Riccio, Simona Ghanitab, Stefania Polletta, Valentina Annesi, Domenico Antonaci, Andrea Rago, Michele Carlo Schiavi, Vincenzo Spina, Rocco Rago

**Affiliations:** ^1^ Reproductive Physiopathology and Andrology Unit Sandro Pertini Hospital, Rome, Italy; ^2^ Obstetrics and Gynecology Unit ASL Frosinone, Frosinone, Italy; ^3^ Protection of Maternal and Child Health Unit, Rieti, Italy

## Abstract

Infertility is increasing worldwide, as well as in Italy, and fallopian tube pathology represents one of the most impacting causes of infertility for multiple women. Indeed, tubal patency assessment is a crucial step in medical evaluation for women attending an in vitro fertilization (IVF) center. Currently, different methods for tubal investigation are available, such as chromosalpingoscopy, hysterosalpingography (HSG), and hysterosalpingo-contrast sonography (HyCoSy). This diagnostic exam is performed by ultrasonography and an air-water-based contrast agent represented by air-water, or foam solution (HyFoSy). An additional side benefit of these assessment tests is a fertility-enhancing effect, thanks to a positive effect defined as “tubal flushing,” which in current literature is more strongly associated with HyFoSy with respect to HyCoSy. In this report, we present a case of a 34-year-old woman presented to our attention at the Reproductive and Physiopathology Unit of Sandro Pertini Hospital, Rome, in 2023, with unexplained infertility for 3.1 years of free sexual intercourse with a partner who did not report sperm abnormalities. Subsequently, in this exam, the woman spontaneously conceived in the same menstrual cycle that the 4D-HyCoSy was performed, without any additional fertility enhancement interventions. In this case report, we also include an updating review of the current literature regarding the insurgence of spontaneous pregnancy after this technique in order to explore the physiopathological and etiopathogenetic mechanisms underlying the achievement of spontaneous pregnancy and to confront our case with other recent works published. According to our clinical experience and the current literature, 4D-HyCoSy is the easiest, safest, and cheapest diagnostic exam for investigating tubal patency, which can lead to medical success in some cases of “unexplained infertility” as the achievement of a spontaneous pregnancy.

## 1. Introduction

Couple's infertility is one of the most impacting diseases of the new millennium in our society and can be defined as a pandemic. Indeed, it is increasing worldwide, as well as in Italy, where at least 1/7 couples of childbearing age are infertile and refers to more than 360 Italian reproductive centers [1, 2]. In several cases, the etiology of infertility is defined as unexplained (25%) or can be caused by male factors, but in one-third of the cases, it is caused by female factors such as advanced age and poor ovarian reserve, endometriosis, and tubal factors. Specific tubal factors can be caused by acute or chronic salpingitis, pelvic inflammatory disease, and pelvic endometriosis involving tubes, pelvic surgery, previous ectopic pregnancy, and septic abortion. This is one of the most impactful causes of infertility, leading to IVF therapy [3]. For this reason, tubal patency represents a crucial evaluation step: an accurate examination is essential in the diagnostic workup of an infertile couple who desires a pregnancy [4].

Nowadays, laparoscopy with dye test, usually represented by chromopertubation, represents a gold standard technique but has the disadvantage of being expensive, related to surgical risk as well as anesthesia risks and a waiting operatory list. On the other hand, Rx-hysterosalpingography (RX-HSG) is a widely accepted procedure for the assessment of tubal patency and uterine cavity, but it shows some limitations [5]. It involves ionizing radiation and the direct injection of an iodinated contrast agent into the uterine cavity, which can cause toxic reactions, and this technique does not give information regarding the ovaries or the external profile of the uterus [6].

On the other hand, hysterosalpingo-contrast sonography (HyCoSy) is an ultrasound-based imaging technique that allows an accurate evaluation of tubal conditions through the dynamic acquisition of real-time images of the uterine cavity and tubal patency [7, 8]. It can be performed by contrast media represented by air-water or foam solution (in this case is defined as HyFoSy, which is in recent years one of the most used and accurate techniques for tubal patency investigation). Indeed, in a recent study, tubal evaluation was conducted between two groups (HyFoSy vs. HyCoSy) and laparoscopy with a dye test. Results demonstrated that the HyFoSy tubal evaluation group was concordant with laparoscopic results in 94.4% of cases, with a sensitivity of 87.5% and a specificity of 100%, whereas in the HyCoSy group, concordance occurred in only 57.8% of examinations, with a lower sensitivity of 50% and a specificity of 66.6% [9]. Despite this fact, the concordance between HyCoSy and RX-HSG was 89.6% in the diagnosis of tubal patency and has proved to be as reliable as laparoscopic techniques in the assessment of tubal patency and uterine morphology by overcoming such major drawbacks as hospitalization, radiation exposure, anesthesia, and use of iodinated contrast media [10].

HyCoSy can be performed by all the reproductive medicine specialists, after a short learning curve, as the first step for evaluating infertile women <40 years old attending a reproductive center. Nowadays, the 4D-HyCoSy investigation is considered safe, well tolerated, rapid, easy to perform, inexpensive, and more accurate with respect to the 2D exam [11, 12]. Therefore, several studies demonstrate an increase in the spontaneous pregnancy rate after HyCoSy, probably due to a positive effect defined as “tubal flushing,” in which several hypothesized mechanisms are involved, but, until now, this aspect is still controversial and debated [13]. A recent Cochrane analysis suggests that tubal flushing as oil-soluble contrast media, in specific foam instillation, may increase the chance of pregnancy and live birth compared to no intervention, and on the contrary, data regarding other contrast media, as an air-water solution, were inconclusive due to inconsistency and lack of statistical power [14, 15]. The aim of this report is to present a case of a woman afferent to our reproductive center who had a spontaneous pregnancy in the same menstrual cycle after 4D-HyCoSy using air-water contrast media. We also present an updating review regarding the possible physiopathological and etiopathogenetic mechanisms involved in the insurgence of spontaneous pregnancy, reporting recent studies published regarding 4D-HyCoSy and spontaneous pregnancy, in order to confront our case with the recent literature and explore this fascinating field.

## 2. Case Report

**Figure 1:**
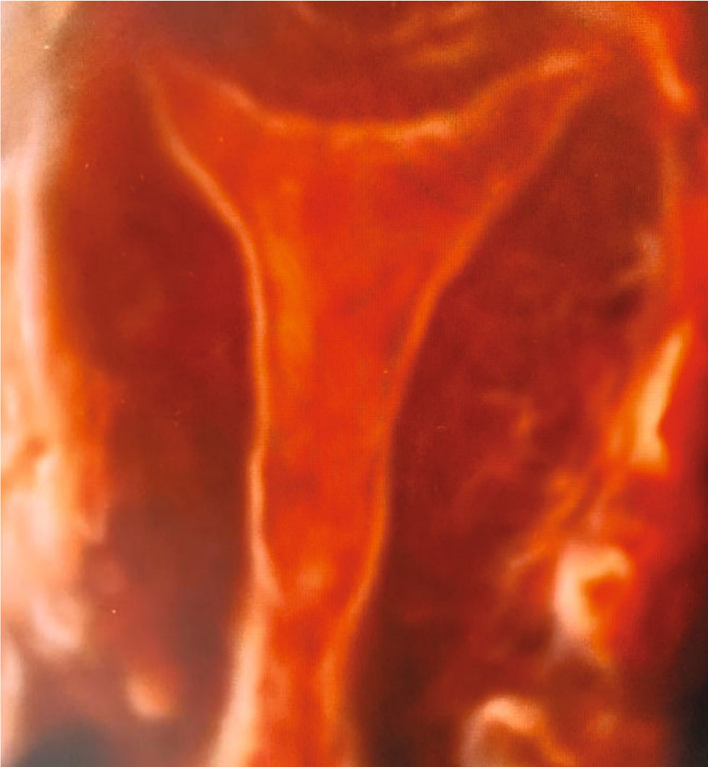
Ultrasound image of the uterine cavity and onset of the tubes, represented thanks to the auxilium of 3-4D software of the ultrasound machine during the HyCoSy exam.

We present a case report of a 34-year-old woman, with a history of 3.1 years of unexplained primary infertility, performing a 4D-HyCoSy at our reproductive center in Sandro Pertini Hospital, Rome, Italy, in March 2023. The patient was in good condition and presented a regular menstrual period of 28 days, a normal value for her age of AMH of 2.6 ng/ml, and an antral follicular count (AFC) of 14. Moreover, she was not affected by endometriosis or adenomyosis, as evaluated by our staff through an accurate anamnesis and transvaginal ultrasound (TVS). The male partner presented a normal sperm analysis, according to the last WHO classification [16]. Before the examination, the patient performed a cervical-vaginal swab that resulted in negative. The exams were performed on the 12^th^ day of the menstrual cycle, according to the standard technique described by Savelli et al. [17]. During the TVS examination, a 3-4 reconstruction of the uterine cavity was performed, which showed a regular external and internal profile without suspicious polyps, myomas, or other intracavitary pathologies (Figure 1). In addition, a dominant follicle of 17 mm in diameter was appreciated in the right ovary. Both tubes were visualized during the exam, and the contrast medium spread bilaterally and regularly, showing tubal patency (20 ml of a solution of air/water was utilized). At the end of the procedure, the patient was monitored in our visiting room for 15 minutes, and no side effects, such as vasovagal reactions, were observed. After the exam, the patient was invited to have free sexual intercourse with the partner without any additional therapy, except for folic acid integrations, which she had assumed daily for 2 months. Around 20 days after the exam, in April 2023, the patient performed a blood pregnancy test that resulted in positive (beta-HCG value of 1420 mUI/ml), and at 6 weeks of amenorrhea, an ultrasound was performed that confirmed a viable intrauterine pregnancy. In December 2023, after a regular and physiologic course of the pregnancy, the woman spontaneously delivered at 39 weeks of gestation, giving birth to a male baby of 3250 grams in good condition.

## 3. Discussion

Our case report presents a real clinical experience of the achievement of spontaneous pregnancy after 4D-HyCoSy, and the intriguing fact is that spontaneous conception was achieved immediately after the exam. Different hypothetical mechanisms are involved regarding this possible therapeutic role and may involve tubes, endometrium, and peritoneum, as reported by a recent work that investigated the role of oil contrast media during HSG [18]. We hypothesize that all those factors can be similarly involved in the tubal flushing effect using the air-water contrast medium.

- The tubes: a mechanical “washing” action performed by the contrast media, such as an oil solution or an air-water solution, permits the removal of mucus, adhesions, or residual material from inflammatory processes. These adhesions may not completely occlude the tubal lumen but could hinder conception or embryo transport. Furthermore, the contrast medium could improve ciliary activity and motility, which are primarily involved in gamete and embryo transport [19].
- The endometrium: increase in endometrial receptivity thanks to an immunobiological action of the contrast medium in modulating endometrial leukocytes. It is possible that the endometrial leukocyte populations of infertile women may be altered, and furthermore, there is evidence that the natural killer lymphocyte cells present in the endometrium play an important role in the initial development of pregnancy [20]. Indeed, in the study of Li et al., the endometrial characteristics, including endometrial thickness and blood flow distribution pattern, were measured twice by transvaginal Doppler ultrasonography in the preovulatory phase before and after the HyCoSy examination. The authors reported that patients performing HyCoSy showed improved endometrial blood flow distribution, indicating that the technique may improve endometrial perfusion and have a therapeutic effect on improving spontaneous pregnancy among women with unexplained infertility [21].
- The peritoneum: the modulation of peritoneal macrophages may have a positive role. Indeed, lipid-soluble contrast agents, as well as air-water solutions, have shown the ability to alter the production of interleukins and prostaglandins by macrophages and to modulate peritoneal macrophage activity and the inflammatory response. Additionally, a recent study has also shown modulation of the action of dendritic cells and T lymphocytes in the peritoneal cavity, which may contribute to improving fertility outcomes [22].

**Figure 2:**
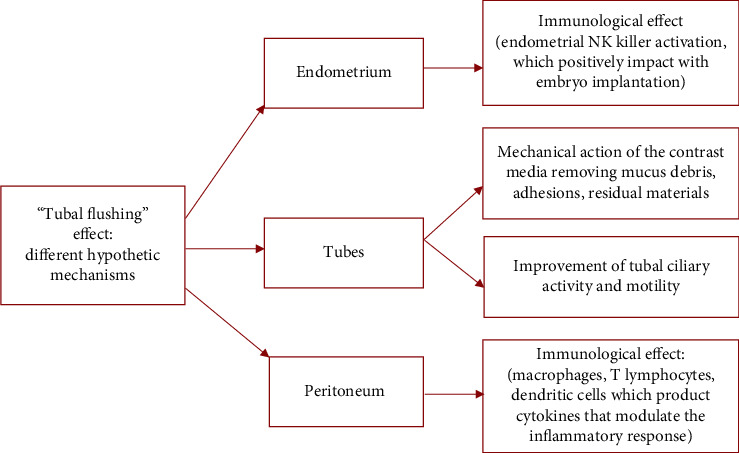
Different mechanisms hypothesized during the HyCoSy exam through air-water insufflation. The contrast medium generates the “tubal flushing effect,” which involves the endometrium, tubes, and peritoneum. Those different immunological/mechanical effects positively impact the insurgence of a spontaneous pregnancy.

Despite the different etiopathogenetic mechanisms explained (reassumed in Figure 2), numerous clinical studies in recent years have highlighted a notable number of pregnancies after this examination. Lindborg et al. compared, through a prospective randomized study, the rate of spontaneous pregnancies in 334 patients, of whom 149 underwent sonohysterosalpingography and 148, on the contrary, did not perform the exam [23]. The primary outcome was evidence of spontaneous pregnancy within 6 months of randomization, while the secondary outcome instead referred to the evolution of the pregnancy. The spontaneous pregnancy rate in the first group was 29.2%, while in the control group, it was 26.5%, a not statistical difference of 2.7% that gives no possibility to confirm the clinical impression of an increase in spontaneous pregnancies. The diagnostic role of sonohysterosalpingography was therefore highlighted, denying its possible therapeutic role for the purposes of spontaneous conception.

In a prospective observational study, Giugliano et al. recruited 180 patients who attended the infertility center of the University of Ferrara during the period 2010-2012. 40 patients (22.2%) achieved pregnancy within 6 months of sonohysterosalpingography, and the average time elapsed before conception was 75 days, but the rate of spontaneous pregnancy was significantly higher in the first 30 days (45%) in comparison with subsequent months (*p* < 0.0005). Such a clear temporal connection strengthens the hypothesis that sonohysterosalpingography can reestablish tubal patency in several cases, allowing spontaneous conception, especially in the cycle in which the examination is carried out [24]. These two studies, which lead to diametrically opposite conclusions, were designed differently. In fact, Giugliano et al.'s study did not include a control group. For this reason, we cannot know whether the spontaneous pregnancy rate detected can be comparable to that of a group with similar characteristics that did not undergo the test, and the authors highlight the close temporal correlation, which can hardly be considered accidental.

Despite those two studies, a recent meta-analysis showed no clear positive effect of the air-water solution on a possible therapeutic role with respect to the oil-based contrast medium, which probably increases clinical pregnancy rates within 6 months and may increase subsequent live birth rates, but evidence on fertility outcomes beyond 6 months is inadequate to draw firm conclusions [25].

Regarding our clinical experience, in a recently published paper, during the period 2019-2021, we evaluated 380 patients performing HyCoSy at our reproductive medical center. Among them, we reported a positive impact of the technique regarding the insurgence of a spontaneous pregnancy of around 30%, and the time to pregnancy (TTP) was 2.1 months [26].

Our previous published results, regarding the possible therapeutic role of HyCoSy, are in accord with Liu et al., in which authors demonstrated that infertile women could succeed in spontaneous conception after 4D-HyCoSy, and expectant treatment of about 8-9 months is reported to be feasible for infertile women whose 4D-HyCoSy findings showed one tube patency or poor patency [27].

In light of those different studies, nowadays, 3-4-dimensional software images, applied with advanced probes, are the best tool equipment for the evaluation of tubal patency and, at the same time, of the uterine cavity and morphology [28, 29].

Moreover, automated 3D volume acquisition permits visualization of the tubes in the coronal view and of the tubal course in 3D space and allows less experienced operators to evaluate tubal patency status easily [30].

To our knowledge, this is the first single case report described in the current literature with the former technique, and the second single case reported in the current literature, where a single case of spontaneous pregnancy is obtained after HyFoSy [31].

Another important element is that the insurgence of a spontaneous pregnancy (TTP) is closely related to the time since the exam was performed. According to another recent work published, the pregnancy rate (PR) was 19.44% within 180 days after HyCoSy and it was significantly higher in the first 30 days (6.35%) [32]. Those results are consistent with our previous work, where the TTP was significantly higher within the first months after 4D-HyCoSy with respect to the subsequent year [26].

According to our clinical experience, this case report, and the recent works published in current literature, this exam permits in some cases of defined “unexplained infertility” to obtain a spontaneous pregnancy. In some cases, HyCoSy avoids IVF tecniques and clinical risks linked to those tecniques such as ovarian hyperstimulation syndrome (OHSS) and obstetrical complications of IVF pregnancies related to different etiopathogenetic mechanisms such as impaired placentation [33, 34]. Therefore, the achievement of a natural spontaneous pregnancy permits saving money for both the national health system and couples because HyCoSy has an estimated cost of around 40 euros for the disposable material, compared to the higher costs of IVF techniques [17]. Also, HyFoSy allows to save economic resources with respect to the HSG exam, from 136 euros to 280 euros, as reported in a recent study conducted along the “Foam trial” [26, 35, 36]. Finally, it is crucial to underline that all those costs are lower with respect to an IVF treatment, and in many developing countries, IVF is not accessible for everyone or not available at all. Indeed, in all these places, HyCoSy may be not only the first-line tubal investigation exam but also a promising alternative to IVF treatments, leading to medical success in some cases of unexplained infertility.

## 4. Conclusion

Today, HyCoSy, thanks to the auxilium of 3–4-dimensional software, represents the first line exam for the investigation of tubal patency before the beginning of IVF techniques. This exam is safe, well-tolerated, simple to perform, and practically inexpensive, and it could play a role in resolving some forms of unexplained infertility. HyCoSy is less invasive and cheaper, especially if performed with air and saline as contrast media, compared with both laparoscopy and RX-HSG, and gives information on the uterine cavity and ovarian morphology, thanks to the 3-4-dimensional ultrasound probe and software equipment that permits instant volume rendering software. Indeed, in an IVF center, 4D-HyCoSy is a crucial step for clinicians to choose and tailor the best reproductive strategy that can support the achievement of a successful pregnancy, which represents a happy event for both couples and specialists involved in medical reproductive medicine. The etiopathogenic mechanism involved in the obtainment of spontaneous pregnancy, mediated by the tubal flushing effect due to the action of the contrast medium, involves different genital districts (endometrium, tube, and peritoneum). Despite the fact that all those physiopathological mechanisms are not completely known, the HyCoSy exam and its application to medical reproductive medicine represent an intriguing topic to explore in further studies.
